# Physicochemical properties and biological effects of quaternary ammonium methacrylates in an experimental adhesive resin for bonding orthodontic brackets

**DOI:** 10.1590/1678-7757-2020-1031

**Published:** 2021-05-03

**Authors:** Tatiana Féres Assad-Loss, Jamille Ferreira Vignoli, Isadora Martini Garcia, Maristela Barbosa Portela, Luis Felipe J. Schneider, Fabrício Mezzomo Collares, Larissa Maria Assad Cavalcante, Monica de Almeida Tostes

**Affiliations:** 1 Universidade Federal Fluminense Programa de pós-graduação em Odontologia NiteróiRJ Brasil Universidade Federal Fluminense, Programa de pós-graduação em Odontologia, Niterói, RJ, Brasil.; 2 Universidade Federal do Rio Grande do Sul Laboratório de Materiais Dentários Porto AlegreRS Brasil Universidade Federal do Rio Grande do Sul, Laboratório de Materiais Dentários, Porto Alegre, RS, Brasil.; 3 Universidade Veiga de Almeida Pós-graduação em Odontologia Rio de JaneiroRJ Brasil Universidade Veiga de Almeida, Pós-graduação em Odontologia, Rio de Janeiro, RJ, Brasil.; 4 Universidade Federal do Rio Grande Pós-Graduação em Odontologia da do Sul Porto AlegreRS Brasil Universidade Federal do Rio Grande, Pós-Graduação em Odontologia da do Sul, Porto Alegre, RS, Brasil.

**Keywords:** Orthodontics, Dental Bonding, Dental caries, Quaternary Ammonium Compounds, Light-Curing of Dental Adhesives

## Abstract

Fixed orthodontic appliances may lead to biofilm accumulation around them that may increase caries risk. This study aimed to evaluate the influence of quaternary ammonium methacrylates (QAMs) on the physicochemical properties, cytotoxicity, and antibacterial activity of adhesive resins for orthodontic purposes.

**Methodology:**

A base resin was prepared with a comonomer blend and photoinitiator/co-initiator system. Two different QAMs were added to the base adhesive: dimethylaminododecyl methacrylate at 5 wt.% (DMADDM) or dimethylaminohexadecyl methacrylate (DMAHDM) at 10 wt.%. The base adhesive, without QAMs, (GC) and the commercial Transbond™ XT Primer 3M (GT) were used as control. The resins were tested immediately and after six months of aging in the water regarding the antibacterial activity and shear bond strength (SBS). The antibacterial activity was tested against Streptococcus mutans via metabolic activity assay (MTT test). The groups were also tested for the degree of conversion (DC) and cytotoxicity against keratinocytes.

**Results:**

The resins containing QAM showed antibacterial activity compared to the commercial material by immediately reducing the metabolic activity by about 60%. However, the antibacterial activity decreased after aging (p<0.05). None of the groups presented any differences for SBS (p>0.05) and DC (p>0.05). The incorporation of DMADDM and DMAHDM significantly reduced the keratinocyte viability compared to the GT and GC groups (p<0.05).

**Conclusion:**

Both adhesives with QAMs showed a significant reduction in bacterial metabolic activity, but this effect decreased after water aging. Lower cell viability was observed for the group with the longer alkyl chain-QAM, without significant differences for the bonding ability and degree of conversion. The addition of QAMs in adhesives may affect the keratinocytes viability, and the aging effects maybe decrease the bacterial activity of QAM-doped materials.

## Introduction

Fixed orthodontic treatment leads to significant caries risk due to biofilm accumulation around the appliance components and the challenges to achieve a reliable oral hygiene.^1^^–^^5^ During orthodontic bonding procedures, the excess of adhesive is invariably left on the tooth surface at the bracket-enamel interface. This adhesive excess may be a site for rapid attachment and growth of oral microorganisms such as *Streptococcus mutans*.^6^ Demineralization occurs when the oral environment pH favors calcium and phosphate ions out of enamel, and early lesions appear clinically as opaque white spots caused by mineral loss.^1^ White spot lesions (WSL) are one of the most common adverse effects of orthodontic treatment,^1^^,^^5^ occurring in almost 50 % of patients during the first year of treatment^4^ and may negatively affect dental esthetics.^1^ Unfortunately, preventive approaches during orthodontic treatments have not been effective, and the occurrence of WSL is positively and significantly associated with the duration of the therapy.^1^^,^^7^

Antibacterial materials could be a promising alternative to biofilm control in orthodontics patients.^8^^,^^9^ The effectiveness of different antibacterial materials added to adhesives has been evaluated *in vitro,* ^8^^,^^10^^–^^13^ *in situ*,^10^ and *in vivo*,^9^ showing positive results to decrease biofilm viability. However, the duration of the antibacterial effect of resins remains uncertain. Among the antibacterial agents that have been tested, the incorporation of methacrylate monomers derived from quaternary ammonium salts has increasing attention. Quaternary ammonium methacrylates (QAM) present antimicrobial mechanisms by disturbing the electrical balance of bacterial membrane through their positively charged molecules with the negatively charged compounds on the bacteria surface. This event leads to bacterial membrane disruption and cell death.^13^^–^^16^ Besides providing antibacterial activity, the incorporation of antimicrobial agents should not alter dental material physical properties.^15^^,^^16^ For orthodontic purposes, the shear bond strength (SBS) between the adhesive and the bracket should not be affected.

However, the literature is divergent regarding the most appropriate QAM and its concentration to be added in adhesive resins for bonding orthodontic brackets.^11^^,^^12^^,^^17^ Previously, the physical properties and antimicrobial activity of two QAM – dimethylaminododecyl methacrylate (DMADDM) and dimethylaminohexadecyl methacrylate (DMAHDM) – were tested at two different concentrations (5 wt.% or 10 wt.%).^18^^,^^19^ The longer the carbon chain of the QAM and the higher its concentration, the higher the antimicrobial capacity achieved. However, the physical properties of the resin materials were jeopardized by increasing QAM concentration. DMADDM at 5 wt.%, in turn, led to the lowest polymer degradation after water storage among groups^19^ while maintaining the antimicrobial property.^18^ However, there is no evaluation of SBS nor cytotoxicity of these adhesives. Our study aimed at evaluating the influence of quaternary ammonium methacrylates (QAM) in the physicochemical properties, cytotoxicity, and antibacterial activity of adhesive resins for orthodontic purposes. For this purpose, we evaluated the addition of DMADDM at 5 wt.% or DMAHDM at 10 wt.% in the base resin considering both the immediate and the long-term antibacterial property, along with SBS. We also analyzed the degree of conversion and cytotoxicity against human keratinocytes. The null hypothesis is that QAM addition in the experimental adhesive will not affect physicochemical properties tested and will not promote antibacterial activity and cytotoxic effect.

## Methodology

### Synthesis of antibacterial monomers

Menschutkin reaction was used to synthesize the antimicrobial monomers, as reported^14^^,^^20^ and previously published.^18^^,^^19^ A tertiary amine and an organo-halide were added to this reaction, in equal amounts (60 mmol), into a round bottom flask coupled to a condenser with 20 mL of ethanol and were refluxed for 24 h. The pure monomer was obtained when the solvent was evaporated in a rotatory evaporator until dryness and so it did not need purification. For each monomer, a different organo-halide was used, since the tertiary amine was always 2-(dimethylamino)ethyl methacrylate (DMAEMA).

### Experimental adhesives formulation

**Figure 1:**
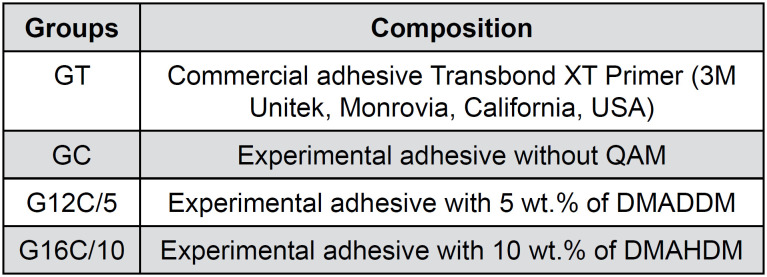
Composition of each adhesive

The adhesive formulation^21^^,^^22^ was the mixture of bisphenol-A-glycidyldimetacrylate (Bis-GMA, Esstech, Essington, Pennsylvania, USA) and 2-hydroxyethylmethacrylate (HEMA, Esstech, Essington, Pennsylvania, USA) at a 55:45 wt.% ratio. To this base resin, 0.5 mol% camphorquinone (Esstech Inc., Essington, Pennsylvania, USA) and 1 mol% EDMAB amine (Sigma-Aldrich, St Louis, Missouri, USA) were added as the photoinitiator/co-initiator system. The synthesized QAMs were added at 5 wt.% DMADDM or 10 wt.% DMAHDM of the total organic matrix weight. The groups were arranged as shown in Figure 1:

### Antibacterial activity

Forty-eight adhesive discs were prepared to evaluate the immediate (T0) and long-term (T1) antibacterial activity (n=6). Metallic molds measuring 2 mm thickness and 1 mm diameter were used. The photoactivation was performed for 20 s on each side of the samples using a light-emitting diode (Emitter B Supra, Schuster Equipamentos Odontológicos Ltda, Santa Maria, RS, Brazil) with 1.200 mW/cm^2^ (measured with a radiometer, RD-7, Ecel, Ribeirão Preto, SP, Brazil). This device was used throughout the whole study. The samples for long-term analysis were stored in distilled water at 37°C for six months. The discs were sterilized with UV light for 1 hour and placed in sterile flat-bottom 24-well plates.^23^ On each well with sterilized adhesive discs, 2 mL of bacterial suspension in Brain Heart Infusion broth (BHI, Difco, Sparks, Nevada, USA) supplements with 2 wt.% sucrose were added. The strain of *Streptococcus mutans* ATCC 25175 (American Type Culture Collection, Fiocruz, Rio de Janeiro, RJ, Brazil) used was cultured in BHI at 37°C for 24 h under low oxygen tension conditions. The bacterial suspension was obtained by adjusting the bacterial growth to 0.5 following the McFarlane seal at 550 nm using UV/Vis spectrophotometer. Next, this suspension was diluted 1:100, and 10 μL of the suspension was added to each well, containing an adhesive disc with 2 mL of BHI broth supplemented with 2 wt.% sucrose. The 24-well plates were rapt for 48 h t 37°C under microaerophilic conditions. Then, 3-[4,5-dimethylthiazol-2-yl]-2,5-diphenyl bromide (MTT, 1 mg/mL in PBS) was added to each well and incubated at the same conditions for 1 h. Subsequently, 1 mL of dimethylsulfoxide (DMSO) was added to each well, and the plates were incubated for 20 min at room temperature. The suspensions were subjected to a spectrophotometer at 540 nm (DU 530, Beckman Coulter Company, Brea, CA, USA). A higher absorbance was indicative of a higher concentration of formazan, which, in turn, indicated a higher metabolic activity of *S. mutans* biofilm.^18^^,^^19^

### Shear bond strength (SBS)

Ninety-six bovine incisors were used to evaluate both the immediate (T0) and the long-term (T1) SBS (n=12). The teeth were fixed with polystyrene resin using a PVC matrix of 20 mm in height and 25 mm in diameter with the vestibular face parallel to the resin base. The labial surfaces of the teeth were polished with water sandpaper of 400 and 600 grain to obtain a flat surface and make a uniform smear layer, rubber brush and pumice. Then, preconditioned with 37% phosphoric acid for a period of 30 s, rinsed with water for 30 s and air-dried. Next, one layer of the adhesive, according to the groups, was applied and air-dried. Brackets (Edgewise, Morelli, Sorocaba, Brazil) were fixed to the centers of the teeth vestibular faces using the same resin cement Transbond XT (3M Unitek, Monrovia, California, USA) for all groups. The resin cement was photoactivated for 10 s on each face of the bracket; thus, the photoactivation time totaled 40 s. The samples were subjected to the SBS test in a universal test machine (EMIC, São José dos Pinhais, PR, Brazil). A knife-edge chisel (0.1 mm) applied at 180° to the tooth labial face was positioned at the adhesive-enamel interface. The force was applied with 0.5 mm/min until the moment the bracket was detached. The results were recorded in MPa.

### Degree of conversion (DC)

The orthodontic adhesives (n=3) were placed on the attenuated total reflectance device of a Fourier transformed infrared (FTIR) spectrometer (Alpha-P; Bruker Optics, Ettlingen, Germany). Each sample was photoactivated for 40 s using the light-emitting diode. The photoactivation was standardized at 1 mm between the light-unit tip and the samples. The same light-curing unit, irradiance, and radiometer were used for all subsequent methodologies. Spectra from FTIR analysis were recorded before and 1 minute after photoactivating each sample with 32 scans and a 4 cm^−1^ resolution. The DC was estimated based on the area of the 1638 cm^−1^ peak (carbon-carbon double bonds of aliphatic chain) and 1608 cm^−1^ peak (carbon-carbon double bonds of aromatic chain) using the following formula:

$$\text{DC}(\%)=100\times \left(\frac{\text{ peak height of cured aliphatic C}=C/\text{ peak height of cured aromatic C}=C}{\text{ peak height of uncured aliphatic C}=C/\text{ peak height of uncured aromatic C}=C}\right)$$

### Cytotoxicity evaluation

To evaluate a possible cytotoxic effect, the adhesives (n=5) were prepared (2 mm thickness and 4 mm diameter) by photoactivation during 20 s on each side. The samples were stored for 24 h in distilled water at 37°C and sterilized with hydrogen peroxide plasma (58%) for 48 min at 56°C.^24^ The cytotoxicity evaluation was performed against human keratinocytes (HaCaT, CLS Cell Lines Service GmbH, Eppelheim, Baden-Württemberg, Germany). One hundred microliters of Dulbecco's Modified Eagle Medium (DMEM, Thermo Scientific, Waltham, MA, USA) with HaCat were inoculated in 96-well plates at 5×10^3^ cells/per well. The plates were kept at 37°C for 24 h. The samples were individually immersed in Eppendorf tubes with 1 mL of DMEM and kept at 37°C for 24 h to prepare the eluates. Then, 100 μL from each Eppendorf tube was placed in contact with HaCat in the wells. Five aliquots of 100 μL were tested from each sample, totalizing twenty-five wells per group. One group was used as control: wells with HaCat and DMEM without eluates. The plates were kept at 37°C for 72 h. After this period, the cells were fixed with 50 μL of trichloroacetic acid (TCA) solution (1 TCA: 1 distilled water) and kept at 4°C for one hour. The plates were washed with running water six times and left at room temperature to dry. Sulforhodamine B (SRB) solution at 0.4% (diluted in acetic acid solution at 1%) was added (50 μL) in each well, and the plates were incubated for 30 min at room temperature. The plates were washed with 1 % acetic acid solution four times and dried at room temperature. Trizma solution 10 mM (100 μL) was added and the plates were incubated for 1 h at room temperature. The plates were analyzed at 560 nm to obtain the absorbance value from each well. The cell viability was normalized against the viability of cells in wells without eluates (control of the test) and expressed in percentage of viability.^24^

### Statistical Analysis

The data were analyzed using the software SigmaPlot, version 12.0 (Systat Software, San Jose, CA, USA). Data distribution was evaluated using the Shapiro–Wilk test. Data from the antibacterial activity and SBS were analyzed using two-way ANOVA and Tukey's test for post-hoc analysis considering the two factors: “time” and “orthodontic adhesive”. The DC and the cytotoxicity were analyzed using one-way ANOVA and Tukey's test. All analyses were performed at a 5 % significance level.

## Results

**Figure 2:**
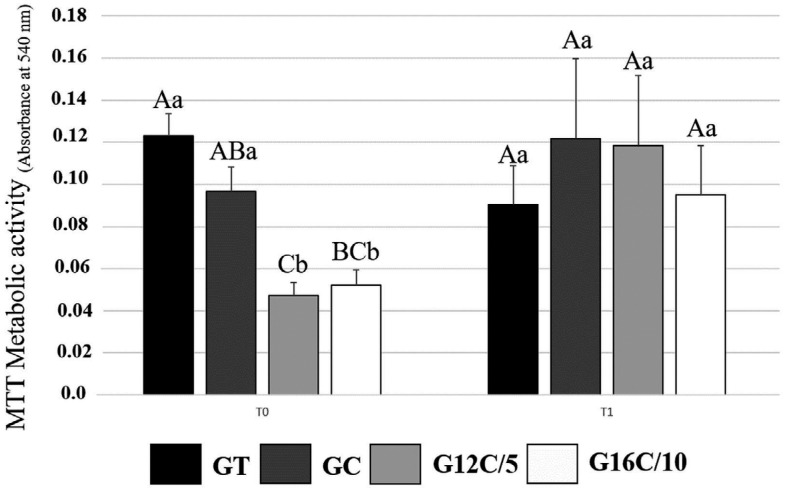
Results of antibacterial activity of the orthodontic adhesive resins. The values of optical density immediately (T0) and after six months of aging in distilled water (T1) are expressed in mean and standard deviation of absorbance values. Different capital letters indicate statistically significant differences among the materials within the same time (p<0.05). Different small letters indicate a statistically significant difference for the same adhesive, comparing the results of different times (p<0.05)

Figure 2 shows the results of antibacterial activity for the orthodontic adhesive resins. The interaction between the variables “time” and “orthodontic adhesive” indicated a statistically significant level at p<0.05. The effect of different levels of “orthodontic adhesive” depended on what level “time” was present. The groups containing DMAHDM or DMADDM showed around 60 % lower metabolic activity compared to GT in T0, with a statistically significant difference between DMADDM and GT to GC (p<0.05), without statistical difference between DMADDM and DMAHDM (p>0.05). After six months of aging in distilled water (T1), the biofilms formed on the samples with DMAHDM or DMADDM showed higher metabolic activity compared to the immediate analysis (T0) (p<0.05). In T1, there was no statistically significant difference among groups (p>0.05).

**Figure 3:**
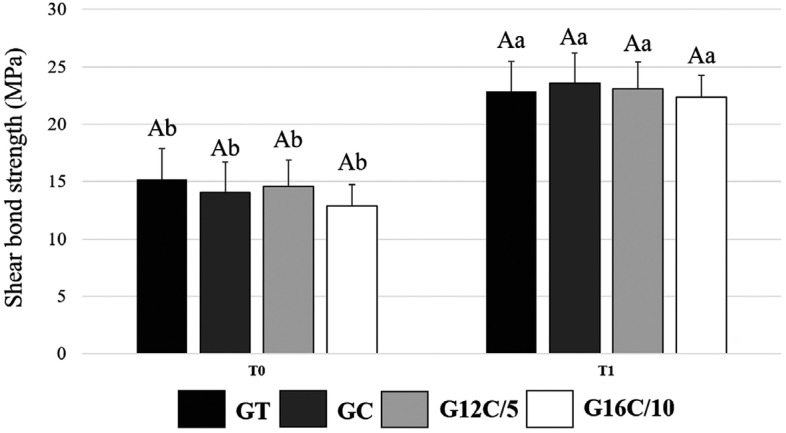
Results of shear bond strength (SBS) immediately (T0) and after six months (T1) of aging in distilled water. The values of SBS are expressed in mean and standard deviation of MPa. Same capital letters indicate no statistically significant differences among the materials within the same time (p>0.05). Different small letters indicate statistically significant difference for the same adhesive comparing the results of different times (p<0.05)

Figure 3 shows the results of SBS immediately (T0) and after six months (T1) of aging in distilled water. In T0, the values ranged from 12.9 (±1.9) MPa for G16C/10 to 15.2 (±2.7) MPa for GT. In T1, the values ranged from 22.3 (±2.7) MPa for G16C/10 to 23.6 (±4.3) MPa for GC. There was no statistical difference among groups in T0 (p>0.05). Moreover, no differences were found among groups in T1 (p>0.05). All groups had an increased value of SBS between T0 and T1 (p<0.05).

Table 1 shows the results of DC of the orthodontic adhesive resins. The values ranged from 72.2 (±1.7) % for G12C/5 to 78.6 (±0.5) % for G16C/10. There was no statistical difference among groups for the DC (p>0.05). Table 1 also shows the results of cell viability. The values ranged from 102.1 (±5.0) % for GT to 12.5 (±3.4) % for G16C/10, with statistically significant differences among groups (p<0.05). G16C/10 showed the lowest value among the adhesives, which was statistically lower than G12C/5 (43.0±9.4 %).

**Table 1:** Mean and standard deviation values of degree of conversion (DC) and cell viability of the orthodontic adhesive resins

Groups	DC (%)	Cell Viability (%)
GT	73.9 (±0.7)^A^	102.1 (±5.0)^A^
GC	76.2 (±0.8)^A^	79.4 (±5.1)^B^
G12C/5	72.2 (±1.7)^A^	43.0 (±9.4)^C^
G16C/10	78.6 (±0.5)^A^	12.5 (±3.4)^D^

Same letters indicate no statistical difference among groups in the same column (p>0.05).

Different letters indicate statistical difference among groups in the same column (p<0.05).

The cell viability was normalized against the viability of cells in wells without eluates (control of the test) and expressed in percentage of viability.

## Discussion

WSL around brackets are a common complication of treatment with ﬁxed orthodontic appliances.^4^^,^^7^ Due to its sealing capacity and antibacterial activity, the application of an adhesive containing QAM may be a promising strategy for the management of WSL. This approach may assist in preventing caries lesions in non-cooperative patients.^11^^,^^12^^,^^25^ In our study, the addition of QAM did not change the physicochemical properties evaluated, and antibacterial activity was achieved in comparison to the commercial orthodontic adhesive. However, cytotoxic effect against human keratinocytes and decreased antibacterial activity were observed after six months of water aging.

An excess adhesive flash promotes a rough site susceptible to the attachment and growth of *S. mutans*.^6^ However, antibacterial polymerized adhesives could inhibit bacteria attachment on enamel margins of the bracket,^13^ which may be especially useful for the site with an excess of adhesive. DMAHDM is a QAM with an alkyl chain length composed of sixteen carbons. Previous studies showed that this QAM exhibits a higher antibacterial effect comparison to DMADDM.^13^^,^^15^ There were no significant differences when the group of 5 wt.% of DMADDM was compared with 10 wt.% of DMAHDM, which differs from our previous study.^18^ Although there was no statistically significant difference between G16C/10 and the control groups in T0, the percentage difference was high (around 60 % compared to GT), and there was no statistical difference among both adhesives containing QAM. In our study, we used MTT assay to evaluate the antibacterial activity. In this test, the reduction of tetrazolium salts to formazan is associated with microorganism metabolic activity. A higher absorbance in the MTT assay suggests a higher formazan concentration and higher metabolic activity. However, this test does not measure cell viability, such as colony-forming units assay. As an initial test to compare the antibacterial activity immediately and over time, it is interesting to use MTT because the pathogenicity of *S. mutans is* related to its metabolic activity and acid production.^26^ Further studies to evaluate the developed materials via colony-forming unit tests would assist in understanding the antibacterial effects.^25^

After water aging, the effect was lower, and no difference was observed among groups. According to Rego, et al.^18^ (2017) the composites containing QAM showed antibacterial activity after brushing simulation. However, there was an increase in viable biofilm after brushing and polishing, suggesting that the antibacterial effects of composites with QAM decrease over time.^18^ The effect of water in the chemical and structure of the polymer network is essential to understand the possible behavior of dental materials in the oral environment.^27^ Besides the aging performed in our study, the effect of degradation was previously demonstrated to reduce the surface hardness, increase sorption and solubility, and hygroscopic expansion.^19^ The leaching part of the QAM from the cured adhesive surface may explain the weakened effect of antibacterial activity after water-aging in our study.^13^ Even with methacrylate groups, part of the QAM may not have copolymerized the resin and was lost during the storage.

There is no standard method for assessing the SBS of brackets to enamel since the loads applied in clinical settings on brackets are dynamic and complex.^28^ A mixture of shear and tensile loads are applied to brackets *in vivo,* and they cannot be perfectly simulated *in vitro*. The forces during chewing are abrupt and impose a high risk of damage to the enamel when compared with the force applied during the SBS test.^28^^,^^29^ However, this is the most widely used assay to assess bonding effectivenesss of orthodontics.^8^ In our study, the SBS results showed no differences among groups, indicating that the addition of the QAM did not affect the mechanical property of the material, corroborating with previous studies.^28^^,^^30^^,^^31^

In the immediate SBS, there was no difference among groups. Interestingly, all adhesives showed higher SBS outcomes after six months. The rationale for that may be delayed in the polymerization reaction when the specimens were stored in warm distilled water over time.^32^ The addition of DMADDM at 5 wt.% also resulted in less degradation for the composite in a previous study.^19^ Besides the non-difference observed for the SBS among groups in the immediate or in the long-term analysis, the DC of the orthodontic resins also did not change with the addition of QAM. This property is related to the chemical and mechanical properties of the polymers, such as diametric tensile strength, compressive strength, hardness, flexural modulus, and strength.^27^ In our study, all groups showed DC similar to commercial^33^ and experimental^34^ adhesives reported in the literature. The higher DC (up to 70 %) observed for all groups may have assisted in the achievement of reliable SBS over time since the higher the DC, the higher the mechanical properties and hydrolytic stability of adhesives.^34^

Cytotoxicity of the adhesives against keratinocytes was evaluated because these cells are present in the oral mucosa. Viable cells were identified using the SRB dye test. Compared to the MTT assay, which is another colorimetric test and an ISO-indicated method. The SRB test has better predictive power by marking viable cell proteins and not relying on cell metabolism for MTT assay. In our study, the cell viability was lower than ISO requires (inferior to 70 % is considered a cytotoxic material when the MTT test is applied), indicating that, even with copolymerizable QAM, probably some of these components may leach from the resin matrix. However, unlike most studies, we used pure eluates on the cells for 72 h without further dilution. Moreover, the samples used had a large area in contact with DMEM for 24 h to produce the eluate, increasing the chances of forming a DMEM with more leached compounds from the specimens and increasing cytotoxic effects. Quantifying the leached compounds of QAMs from the resins was beyond the scope of this study; however, future investigations are encouraged to analyze the leached compounds over time and their correlation with antibacterial and cytotoxic activity.

Even though we evaluated the cytotoxic potential of the adhesives with the highest possible challenge against the cells, careful application on tooth surfaces is recommended. However, the amount of adhesive used in a bracket bonding is only one thin layer in contrast to 4 mm × 2 mm of adhesives discs tested in the study. Other authors tested QAM against fibroblast and odontoblasts, and there were no differences in cytotoxicity compared to commercial adhesives.^15^ On the other hand, our experimental adhesive without QAM^21^^,^^22^ also showed a decrease in cell viability, suggesting that other experimental adhesive components could be involved in this process and lead to cytotoxicity.

## Conclusions

In our study, the effects of two different QAMs in an adhesive for orthodontic purposes were evaluated. DMADDM at 5 wt.% and DMAHDM at 10 wt.% were incorporated into an adhesive blend. The physicochemical and antibacterial properties, besides the cytotoxicity, were analyzed. In short, both adhesives with QAMs showed a significant reduction in bacterial metabolic activity, but this effect decreased after water aging. Lower cell viability was observed for the group with the longer alkyl chain-QAM, without significant differences for the bonding ability and degree of conversion. The addition of QAMs in adhesives may affect the keratinocytes viability, and the aging effects may decrease the bacterial activity of QAMs-doped materials.
